# Illustrations of the Successful Treatment of Cleft Palate by the Division of the Levator-Palati, Palato-Pharyngeus, and Sometimes the Palato-Glossus Muscles

**Published:** 1852-07

**Authors:** John Avery

**Affiliations:** Surgeon to Charing-Cross Hospital.


### ARTICLE XV.

*Illustrations of the successful treatment of Cleft Palate by the Division of the Levator-Palati, Palato Pharyngeus, and sometimes the Palato-Glossus Muscles.* By John Avery, Esq., F. R. S., Surgeon to Charing-Cross Hospital.

The operation recommended by Professor Ferguson: its advantages. Dr. Warren's proposal for its performance. Extensive incisions advised by continental surgeons. Cases detailed, in which the operation has proved successful. Suggestion for modification of the cutting-instrument employed. Bloodlessness and little pain, or constitutional disturbance attendant on the operation. M. Sédillot on staphyloraphy. A desideratum attained in adoption of the process pointed out for the cure of cleft palate.

The principal reason for publishing the three following cases of cleft palate, is to show the complete and speedy success which resulted in all of them, after the division of the above named muscles, as recommended by Professor Fergusson, of King's College; and although their number is small, their results yield good testimony in favor of this particular method, the merits of which will be more impartially appreciated in the successful cases of those who are not the inventors of the method than in the cases of the inventor himself. They may also serve to call the attention of the profession again to this able, ingenious, and scientific operation, of the feasibility and utility of which many skillful surgeons still remain very sceptical.

A careful review of all the different modes of uniting the cleft palate, shows that the plan alluded to is the only one founded on a truly scientific basis—on strictly anatomical and physiological grounds—the only one, indeed, offering fixed and precise rules, by which the operator, who has made himself well acquainted with the anatomy of the parts, can, with certainty, and without any extraordinary skill, accomplish the division of all the muscles which tend to pull the disunited parts asunder. This is done in the manner proposed, with the greatest effect, and the least possible mutilation, and in such a position as to produce the least amount of irritation during the after treatment, and also to give the greatest aid, in all other respects, towards keeping the parts in perfect quietude. In consequence of the almost complete temporary paralysis of the muscles thus produced, full advantage can be taken of giving liquid food from the earliest moment, a proceeding so much insisted on by Sir Philip Crampton, and scarcely second in importance to anything connected with the operation. The only operator who might appear in any way to have anticipated the merits of this proposal, is Dr. I. M. Warren, who made some incisions which had partially the effect of those made by Mr. Fergusson; but any one can convince himself that the true principles of the operation were not understood by Warren, until he had become acquainted with Mr. Fergusson's paper, when he discovered how near he had accidentally been to the truth, without having been acquainted with, or at least having explained, the real reason for his incisions. Before he had seen the paper alluded to, he says,—"*If* the fissure is wide, and this (drawing the flaps together) cannot be effected, I have found the following course to be invariably followed by success. The soft parts being forcibly stretched, a pair of long, powerful, French scissors, curved on the flat side, are carried behind the anterior pillar of the palate: its (the palate's) attachments to the tonsil and to the posterior pillar are now to be carefully cut away, on which the anterior soft parts will at once be found to expand, and an ample flap be provided for all desirable purposes."

The conjunction in italics, shows clearly that Warren disregarded the action of the muscles entirely, if the parts could be brought together, which is relinquishing the very essence of the new method. And when he judges the incision necessary, the mere separation of the attachments of the palate to the tonsil and posterior pillar, is only a very small part of the divisions lately proposed. It is only after he becomes acquainted with this proposal that he directs (in the *American Journal of Medical Science*, vol. xli, 1848, p. 330,) "to cut away the posterior pillar of the palate with strong curved scissors, and *continue*

*the dissection behind the soft palate until the latter yields, and allows itself to be drawn across the chasm,*" and here, again, only when the "halves are too small to be easily brought into contact." But the true principle—and this ought never to be lost sight of—is, not only to allow the halves to be brought easily into contact, but to prevent, as much as possible, all muscular action which could draw the parts asunder after the operation; and the less completely this principle is followed out, the more, I firmly believe, will be required to be done afterwards to remedy partial failures, which would not happen if the directions were rigidly adhered to. This is pretty well exemplified in a case which lately formed the subject of a clinical lecture, by Mr. Quain, published November 10, 1849. That gentleman seems still inclined to lay more stress, in his operation, on destroying the "natural elasticity" of the palate, and not clearly to admit the necessity of paralyzing the muscles, wherever they might act afterwards in drawing the parts asunder; for, he says, he is "not satisfied of the necessity of cutting the palato-pharyngeus and palato-glossus." The success of this case does not appear to warrant the counsel he has given his pupils, to leave out one of the principal parts of the method which has already proved so successful. The fissure was described as being simply in the soft palate, and in all respects favorable for the operation, yet, when the ligatures were taken out, two largish apertures remained open, and were only gradually diminished by the frequent application of nitrate of silver, and ultimately required the application of the actual cautery to close them. Indeed, an inference might fairly be drawn, that the success of the operation had been jeopardized, in consequence of the necessary division of the muscles not having been made. Nor can I agree with Mr. Quain in his advice to allow the parts to remain two hours after the operation before finally closing the sutures, "as the oozing may cause sickness," for I cannot imagine anything more calculated to produce sickness than the renewal of the bedevilment and discomfort of this very disagreeable operation, instead of at once concluding all the steps, giving the patient some comforting food, and placing him in bed.

Extensive incisions have been advised at various times, by Dieffenbach, Mettauer, Pancoast, and others, and no doubt, success will follow in many of these cases. My friend, M. Sédillot, the distinguished professor of military surgery, at Strasbourg, has lately succeeded admirably in uniting the simple fissure of the soft palate, in two cases, where he separated the posterior pillar of the palate, in the manner advised by Warren, and at the same time made an extensive lateral incision through the whole of the thickness of the velum, as practiced by Pancoast. He has also invented some very ingenious instruments for passing the sutures. But the aim of the surgeon should be to attain his object with as little mutilation as possible, and to simplify, instead of complicating the apparatus; and I can bear testimony to the perfect ease with which all the instruments advised by Mr. Fergusson, can be used; and how perfectly they answer the purpose they are intended for. I regret, that the able surgeon of Strasbourg, who was fully aware of the new method, had not given it a fair trial, as I feel assured, he would then have furnished us with additional evidence in favor of the more simple and scientific proceeding.

*Case* 1.—Mr. H——, aged twenty-two, a stout and florid looking young farmer, apparently in good health when he presented himself with cleft palate. The cleft commenced almost immediately behind the hard palate, and divided the velum and uvula into two equal halves. When irritated, the uvula and velum were so forcibly drawn upwards and outwards, that they became scarcely perceptible. When he attempted to swallow, the two sides nearly touched in the mesial line. He bore handling the parts well. His gums were spongy; his teeth were covered with tartar; his throat was red, relaxed, and covered with thick mucus. The wind being easterly at the time, and the weather very cold, he was directed to get his teeth attended to by a dentist, to take a quinine mixture, to use an alum gargle, and return to town when the weather was milder. He returned in May, and on the 20th, the operation was performed, in accordance with the directions given in Mr.

Fergusson's paper. First, the prominent ridge formed by the levator-palati, which is well seen in relief, coming from behind the opening of the eustachian tube, and widening as it descends, was divided with the knife curved on the flat, just where it passed into the velum, and from above downwards, so freely, that the edge of the knife could be seen moving above the mucous membrane of the under surface of the soft palate. This division was made first, without drawing the soft palate inwards, and completed afterwards, by extending it gently towards the mesial line, whilst the incisions were made. The posterior pillars of the fauces were then drawn out with forceps, and divided with a bistoury; they were afterwards pinched up, and further divided by a pair of curved, blunt-pointed scissors. The flaps now hung quite flaccid, and could be brought without difficulty to meet each other. It could not be said, they had no movement at all left in them, but they were nearly disabled, and more particularly at the posterior part of the velum and uvula. No difficulty at all occurred after this, in transfixing the velum at about a line from its free edge, and commencing quite at the anterior angle of the cleft, with a small pointed scalpel, and paring the edges to the very end of the uvula, without once taking the scalpel out, the parts being so perfectly under command from the previous incisions. Five sutures were then passed, by means of the simple handled nævus needle, slightly curved on the flat, proceeding from before backwards, and they were tied by the admirable knot described in the paper alluded to, and immeasurably superior, I believe, to all the devices hitherto invented for the purpose. In this case, it was not necessary to divide the anterior pillars of the fauces. The parts hung without the slightest appearance of tension. The patient bore the operation with great fortitude, and did not complain of much exhaustion: and it seemed to me, in this as well as in the succeeding cases, that the latter steps of the operation were much more supportable after the incisions had been made. He had a little weak barley-water to wash his mouth with, and he swallowed it without irritation. At eight o'clock P. M., he felt very comfortable, took a small cupful of beef-tea, and had an injection of beef-tea, a wine-glass of sherry, and a drachm and a half of laudanum. Barley-water was placed by him to drink occasionally during the night. The remaining treatment and progress of the case are very simply detailed. He took generally, a little beef-tea, and a cupful of gruel with sherry in it twice a day, and also had an enema with a wine-glass of sherry in it, morning and evening, for the first six days, when he had mock-turtle soup, and went into the park. On the ninth day he had chicken and wine and water. On the twelfth, meat and porter, and went to see the Thames tunnel; and on the thirteenth he went home, with the part free from all danger of separation. His system was scarcely effected during the cure; his pulse, which was remarkably low for his time of life, never having been higher than 57 to 60, and sometimes as low as 48. He was a little sick on the third night, without the slightest effect on the ligatures. Forty-eight hours after the operation, the first and third ligatures were removed; on the third day, the fifth; on the fourth day, the second; and on the sixth, the fourth, which still held firmly when it was removed. After the third day, all fear of separation had nearly subsided. There were small ulcerations where the ligatures had been, and spots along the line of union were not completely healed on the under surface, but they all gradually closed by the thirteenth day, excepting one small place, about which there was no anxiety. The improvement in his speech was very marked as regarded making himself understood, the nasal twang remaining. There had been words which he had previously found it almost impossible to utter intelligibly, and which he could now easily make any one understand. His swallowing was also greatly improved.

*Case* 2.—This case was a patient of my friend Mr. Yearsly, of Savile-row. Mr. Fergusson had, with his accustomed liberality, demonstrated to him the preparation which had been the basis of his discovery. Mr. Yearsly had also witnessed my first operation; I assisted him in his, and had the opportunity of watching the progress of the case, and therefore, can vouch for complete justice having been done to all the steps of the new method. It is unnecessary to enter minutely into the history of this case; for the age, the size, the condition of the patient, the character of the deformity, as well as the exact steps of the operation, the treatment, progress, and termination of the case, were as nearly as possible the same as in that already related, and the union was quite as perfect and immediate, and the result altogether as satisfactory.

*Case* 3.—This case was one of a much more aggravated character; so much so, indeed, that I did not anticipate a complete cure, and undertook the attempt, with the promise of closing the cleft in the soft palate only, and to leave the cleft in the hard palate to Mr. Thomas Bell, who had engaged to close that part effectually by means of an obturator. The result, however, was one of perfect success, with one operation only, and shows, in a proportionately clear light, the great value of the plan adopted. Henry B——, aged seventeen, healthy, but strumous and boy-looking for his age; had just returned from the country, where he had been for a couple of months for the purpose of getting himself into condition. He had hare-lip and cleft palate on his left side at his birth, and I learn from his mother, that the cleft extended originally from the lip, through the alveolar process, to the end of the uvula. This is probable, as there is still a deformity in the regularity of the teeth, one of the incisors being absent entirely, and two others very much squeezed together. The hare lip was united when he was very young. At the time of the operation, the cleft commenced in the hard palate, just behind the alveolar process, and continued directly backwards, dividing the soft palate and uvula into two halves. The division was more to the left than the right, particularly in the hard palate, as the vomer was attached to the hard palate of the right side, and the principal deficiency of bone was on the left side, there being very little breadth of bone in this situation. On the inner edge of the bony palate on this, the left side, there was a firm, roundish, ligament-like band, which was continued into the edge of the fissure in the soft palate. Both sides of the bony palate inclined very much upwards, so as to form an acute angle on each side with the alveolar processes—a form which I believe is not usual in these cases, and which rendered the separation of the mucous membrane from the hard palate a matter of unusual difficulty. Both sides of the soft palate and uvula were pretty thick. The right did not extend so far backwards as the left, so that the two halves of the uvula hung very unevenly in the back of the throat. They almost disappeared at the sides of the fauces, when they were irritated, and approached each other very nearly on every attempt to swallow. The cleft in the hard palate was an inch in length, and the division, at its posterior part, was about three-quarters of an inch wide. The first step of the operation was to divide freely the levator-palati on each side, as in the first case; then the palato-pharyngeus. The flaps on both sides now lay much relaxed, particularly at their posterior part; but, as in the former instance, a slight movement still existed. After this, with the same curved knife used for dividing the levator-palati muscle, the fleshy part of the roof of the mouth covering the bony palate, was completely separated from the bones, in the way recommended by Warren; and then, with a pair of strong, sharp-pointed scissors, curved on the flat, the soft palate was completely separated from the posterior edge of the palate bones, by cutting upwards into the nares, and leaving an entire loose flap on each side, extending from the anterior angle of the cleft behind the alveolar processes, to the end of the uvula. This part of the operation required great care, in consequence of the firm adherence, thinness, and brittleness of the textures covering the bones, as well as from the angle above mentioned, which made it extremely difficult to avoid cutting through the soft parts at the kind of chink near the alveolar processes. This proceeding of Dr. Warren was not effected with the expectation of producing the ultimate closure of the fissure in the hard palate, but merely to allow the anterior part of the cleft in the soft palate to come close, without strain, on the ligatures, and the first ligature was consequently placed in the soft palate, and none in that part of the flaps which had covered the bones, fearing they would cause sloughing, from the peculiarity of the textures already alluded to. There was every reason, however, to believe, afterwards, that if one or two ligatures had been placed on that part of the flaps, union would have taken place in the whole line at the same time, as it did in the soft palate, instead of gradually, as was really the case. Five sutures were placed in the soft palate, in the same order as before; and in consequence of the unequal length of the flaps, they were placed obliquely, in order that when they were tied, the two sides of the uvula should exactly correspond. In endeavoring to bring the flaps together, there was difficulty in making the left side advance sufficiently forward, without too much strain, and at last it was determined to divide the palato-glossus on that side. The difficulty was at once removed; the sutures were tied as in the other case, and the parts brought into the most satisfactory state, those in the hard palate being quite in opposition, although no ligature held them together. The operation lasted nearly an hour, notwithstanding there had been no hitch in any part of it, and every instrument fully answered its end. The only suggestion I would offer, and I do it with some diffidence, is to make that portion of the curved knife which is especially used in cutting, flat, instead of curved, as this particular shape is ill adapted for making a clean cut.

The poor boy bore the operation with great fortitude, and no bleeding occurred in this or the former operations. He passed the first afternoon very comfortably, without irritation or cough. At night his skin was rather hot, and his pulse 90. He slept well. On the next morning, early, he was sick, and vomited a good deal of bilious matter, it was supposed from his having taken some arrow-root made with milk. Some of the matter vomited passed through his nostrils. Afterwards he complained of slight headache. Pulse 90; skin rather warm, but moist. The parts were not the least deranged by the vomiting; and afterwards he went on perfectly well, as regarded his health, enjoyed his food with great relish, and was no farther troubled with irritation, or cough, or sickness. Immediately after the operation, he took thin gruel, which was repeated at pleasure. In the evening he had arrow-root, with three-quarters of a wine-glass of sherry; and at night an enema, composed of beef-tea, one drachm of laudanum, and a glass of sherry. He continued his beef-tea enemata with sherry, night and morning, up to the sixth day after the operation, as well as the arrow-root and wine, with an egg occasionally beaten up in it, or isinglass in beef-tea, and similar food. On the sixth day he had wine and water, and ox-tail soup, thin puddings, and bread and milk. On the ninth day he had a mutton-chop, and, after that, game, or any other simple animal food. Two clear days after the operation, the line of contact was still perfect throughout, and the parts about the uvula, and between the third and fourth ligatures, were apparently firmly united. After the completion of the third day, the second suture was removed, and on the same evening, the third and fifth were also taken away. After the fourth day, an opening in the hard palate was visible to a slight extent, but the first suture immediately behind it held firmly, and, indeed, union seemed to have taken place between the opening and the ligature. After the fifth day, the first ligature was removed, and great anxiety was felt until next day, when all was found firmly held together, then the fourth and last ligature was removed, and all anxiety ceased. The opening in the hard palate enlarged to the extent of more than half an inch, and at one time a little backwards and laterally, as if the whole parts behind were contracting bodily more to the left side than to the right. On the seventh day he spoke a little, but he was not allowed to converse for some days after. He was well enough to leave before the fortnight was completed; and he continued to attend, at first, every two or three days, and then every day, and an attempt was made to produce granulations by the application of a strong solution of cantharides in acetic acid, without effect, so the part was left to itself, as the edges were quite close together. In a couple of months it was impossible to pass anything between them, although I believe they are not absolutely grown together even now.

One can scarcely imagine an operation of any importance more bloodless, or followed by less pain, by less local or constitutional disturbance, than in these three cases. At most, there was a slight attack of sickness, which speedily disappeared. Much of this tranquillity depended, no doubt on the early and continued exhibition of nourishment; but it may also be taken into account that the principal incision—that of the levator palati—is situated in the nares, where the parts are united, and thus removed, as it were, from the action of whatever is swallowed, and consequently not so liable to produce irritation. One cannot compare these cases strictly with those where food was not allowed; but the cases alluded to of M. Sédillot, where food was allowed, and the incisions carried completely through the whole thickness of the palate, abundant hæmorrhage occurred in one; in both it was found necessary to bleed afterwards, and in his second case there was a good deal of pain, local inflammation, and fever. Most of the operators who have succeeded to any extent in these cases have used their own peculiar methods, which have not been found to answer in the hands of others in their own or in other countries; and a method which would succeed, irrespective of extraordinary skill or luck of individual surgeons, was still a desideratum. This fact is difficult to account for. Some explanation of it may be found perhaps in a quotation from M. Sédillot's papers, where he speaks of the success of his own countryman, M. Roux, whose good fortune in these cases has been generally believed in this country to have been extraordinary. "The illustrious inventor of staphyloraphy," says M. Sédillot, "not having published the results of his vast experience on this subject, it is not known what has been the proportion of cures and failures in more than 110 cases, I believe, operated on by him. However, the general opinion is, that success has been the exception, and staphyloraphy has been by degrees abandoned by surgeons." We can scarcely imagine, on this side the channel, the *bonne foi* of any of our own eminent surgeons treated with so much freedom. Yet M. Sédillot must be too well acquainted with what is doing in France not to have some foundation for so decided an opinion. From the happy results of Mr. Fergusson's cases, from those of various other surgeons in London who have employed the same means, and from my own experience, I believe the desideratum of a method, which will, to a considerable extent, be successful wherever it is used, and requiring only a good knowledge of the anatomy of the parts, and a fair amount of surgical skill, has been supplied, by the proposal to divide the levator-palati, the palato-pharyngeus, and, if necessary, the palato-glossus muscles.—*Lancet.*

### EDITORIAL DEPARTMENT.

